# Think of your art-eries: Arts participation, behavioural cardiovascular risk factors and mental well-being in deprived communities in London

**DOI:** 10.1016/j.puhe.2012.05.025

**Published:** 2012-09-01

**Authors:** A. Renton, G. Phillips, N. Daykin, G. Yu, K. Taylor, M. Petticrew

**Affiliations:** a Institute for Health and Human Development, University of East London, Water Lane, London E15 4LZ, UK; b Department of Health and Applied Social Sciences, University of the West of England, Bristol, UK; c London Arts in Health Forum, London, UK; d Department of Social and Environmental Health Research, London School of Hygiene and Tropical Medicine, London, UK

**Keywords:** Art, Creative, Craft, Culture, Physical activity, Healthy eating, Mental health, Wellbeing, Health improvement, Health promotion

## Abstract

**Objectives:**

To investigate the association of participation in arts and cultural activities with health behaviours and mental well-being in low-income populations in London.

**Study design:**

Cross-sectional, community-based observational study.

**Methods:**

Data were taken from the cross-sectional baseline survey of the Well London cluster randomized trial, conducted during 2008 in 40 of the most deprived census lower super output areas in London (selected using the English Indices of Multiple Deprivation). Multiple imputation was used to account for missing data in the Well London survey. Descriptive statistics and regression analyses were used to examine the association between participation in arts and cultural activities and physical activity (meeting target of five sessions of at least 30 min of moderate-intensity physical activity per week), healthy eating (meeting target of at least five portions of fruit or vegetables per day) and mental well-being (Hope Scale score; feeling anxious or depressed).

**Results:**

This study found that levels of arts and cultural engagement in low-income groups in London are >75%, but this is well below the national average for England. Individuals who were more socially disadvantaged (unemployed, living in rented social housing, low educational attainment, low disposable income) were less likely to participate in arts or cultural activities. Arts participation was strongly associated with healthy eating, physical activity and positive mental well-being, with no evidence of confounding by socio-economic or sociodemographic factors. Neither positive mental well-being nor social capital appeared to mediate the relationship between arts participation and health behaviours.

**Conclusion:**

This study suggests that arts and cultural activities are independently associated with health behaviours and mental well-being. Further qualitative and prospective intervention studies are needed to elucidate the nature of the relationship between health behaviours, mental well-being and arts participation. If arts activities are to be recommended for health improvement, social inequalities in access to arts and cultural activities must be addressed in order to prevent further reinforcement of health inequalities.

## Background

The use of arts and creative activity therapies in clinical settings, for a range of medical conditions related to both physical and mental health, is supported by the Department of Health for England.^1–3^ Such interventions can include visual arts, drama, music, movement-based arts and dance, and creative writing. There is robust evidence of their effectiveness, particularly in relation to improving outcomes and prognosis amongst patients with diagnosed mental health conditions.^4–10^

Whilst the benefits of arts and creative activities are well researched and widely used in the clinical medical field,^9,10^ there is less evidence about the benefits of these activities for maintaining health or preventing ill health. However, there is strong advocacy for, and substantial use of, arts and creative activities to address social and cultural drivers of poor health^11^ and for conveying health promotion messages.^12–14^ As yet, there have been few high-quality controlled evaluations of such interventions.^15–20^

The aims of this study were: to assess the prevalence of creative activity participation and cultural event attendance in low-income groups in London, and compare them with the national population in England; to investigate the association of receptive and active participation in arts and cultural activities with healthy eating, physical activity, and mental health and well-being; and to assess the evidence that social capital or mental health might mediate the relationship between arts participation and health behaviours.

## Methods

### Data source

Data from the adult baseline survey of the Well London cluster randomized trial (CRT), ^12^ to examine the association of participation in arts and cultural activities with healthy eating, physical activity, and mental health and well-being in a low-income group. Within the Well London CRT, a pair of census lower super output areas (LSOAs) amongst the 11% most deprived (based on the English Indices of Multiple Deprivation) across London were selected in each of 20 London boroughs. For the purposes of the trial, these pairs were randomized to be in the intervention or control group. This study used data from the household adult survey collected in all LSOAs during 2008. Survey respondents were identified through random selection of 100 households (postal delivery points) in each LSOA, and all consenting adults aged ≥16 years were interviewed. Written, informed consent was obtained from all participants. The baseline survey provides information on: socio-economic and sociodemographic factors, to allow robust adjustment for these potential confounders; social capital indicators, to allow examination of potential mediation, in line with current theories on the impact of arts on health outcomes^21^; and measures of arts participation that allow examination of a potential dose–response relationship.^20^

### Creative activity participation and cultural event attendance questions

Two questions about arts participation and event attendance were included in the Well London baseline questionnaire, adapted from questions in the Taking Part survey, conducted by the Department for Culture, Media and Sport. Table 1 shows the types of creative activities and arts events included in the question response options. Respondents were asked how many of the arts and creative activities they had participated in during the last 12 months, and how many of the cultural events they had attended in the last 12 months; they were asked to select all applicable options. Participation in creative activities such as painting, photography, music and other performance was considered to be ‘active participation’, and this activity is referred to as ‘participation in creative activities’.^20^ Participation in events such as theatre, festivals and films was considered to be ‘receptive participation’, and this activity is referred to as ‘attendance at cultural events’.^20^

### Health behaviours and outcomes

The structured questionnaire used for the Well London baseline survey contained validated measures of: healthy eating [food frequency questionnaire adapted from the Health Survey for England 2008,^22^ used to create a binary indicator for meeting the Department of Health's recommended minimum of five portions of fruit or vegetables per day (‘five-a-day')]; physical activity [International Physical Activity Questionnaire, provides binary indicator of meeting the Chief Medical Officer's recommended five sessions of at least 30 min of moderate-intensity physical activity per week (‘five-a-week')]^23,24^; positive mental well-being (Snyder Hope Scale^25^); and self-report feeling anxious or depressed at time of interview from the EuroQol 5D.^26–28^ Respondents were also asked about general practitioner consultations for anxiety, depression or a mental/nervous/emotional problem in the previous 12 months.

### Social capital indicators

Two social capital constructs were measured in the Well London baseline survey: social networks and social support. The questions were taken from the Office for National Statistics (ONS) social capital harmonized question set.^29^ The social network questions asked how frequently the respondent had contact with relatives, friends and neighbours in person, via telephone or via other electronic communication. Only the questions about friends and neighbours were used in the analysis because they represent the type of social capital that might be acquired through arts participation. The social support questions asked respondents how many people would provide help with: groceries if they were unwell; money for a few days; or emotional support in a crisis.

### National comparison dataset

The prevalence of creative activity participation and cultural event attendance in the Well London adult population was compared directly with data from a nationally representative sample of adults from the Taking Part survey of the Department for Culture, Media and Sport.^30^

### Data analysis

All analyses were undertaken using Stata Version 11.2.^31^ Proportions and means with confidence intervals (CI) based on robust standard errors, to account for LSOA-level clustering (the ONS geographical LSOA), are presented for each sociodemographic characteristic and health outcome. Appropriate survey weights were used in analysis of the Taking Part survey. Fixed effects regression models were used to examine the association of sociodemographic characteristics and arts participation with physical activity, healthy eating and mental well-being, using indicator variables for LSOA to account for clustering in the data. Logistic regression was used for the binary outcomes. Multiple imputation was used to account for missing data in all variables from the Well London baseline survey that were included in the analysis.^32–38^ Ten imputations were completed, with 10 cycles in each imputation. Clustering in the dataset at the LSOA level was accounted for in the imputation model using a fixed effect indicator for LSOA. Results are presented from the analysis of the multiply imputed datasets.

## Results

### Response rates for the well London survey

The survey was completed by 4107 adults. The mean household-level response rate in the baseline survey was 73.3% across the 40 LSOAs (standard deviation 13.9; range 40.5–98.9%). The mean individual-level response rate within responding households was 61% across the 40 LSOAs.

There were no missing data in the questions about participation in creative activities and cultural event attendance. Supplementary File 1 shows the proportion of missing responses for each of the other variables used in the analysis. In total, 2077 adults completed the questionnaire items for all the variables used in the regression modelling, on which the complete case analysis was based.

### Arts activities attendance and participation

Three-quarters of the Well London baseline survey population had either participated in at least one creative activity or attended at least one cultural event in the 12 months before their interview (75.3%, 95% CI 71.8–78.7). This contrasts with 90% (95% CI 89.0–90.1) of adults in the national Taking Part survey who had either participated or attended in the previous 12 months. Participation in creative activities was reported by 68.2% of the Well London population (95% CI 64.4–72.0), 60.5% had attended a cultural event, and 53.5% (95% CI 49.3–57.6) had undertaken both types of activity at least once in the previous 12 months.

There was no substantial difference in creative activity participation or cultural event attendance between men and women in the Well London survey, whereas in the Taking Part survey, more women than men reported participation in a creative activity (men 70.0%, 95% CI 68.2–70.8; women 82.0, 95% CI 81.0–82.9), although attendance at cultural events was similar in the two groups (men 74.9%, 95% CI 73.7–76.0; women 78.2%, 95% CI 77.2–79.1). Individuals in the Well London population who had participated or attended in the previous 12 months were substantially younger than those who had not participated or attended (participation/attendance 35.7 years, 95% CI 34.6–36.7; no attendance/participation 45.9 years, 95% CI 43.8–48.1). There was very little age variation in participation in creative activities in the national Taking Part survey, although attendance at cultural events decreased slightly with age.

There was no notable difference in creative activity participation or cultural event attendance amongst different ethnic groups in the Well London population, whereas in the national Taking Part survey, individuals of Indian, Pakistani or Bangladeshi origin had the lowest levels of creative activity participation (57.5%, 95% CI 52.5–62.5) and individuals of White or mixed ethnicity had the highest levels of creative activity participation (White 77.2%, 95% CI 76.4–78.0; mixed 78.7%, 95% CI 71.3–86.0). There was less ethnic variation in cultural event attendance at the national level.

Well London survey respondents who had no formal qualifications, were unemployed, living in rented social housing or found it difficult to manage on their household income, were much less likely to have participated or attended in the previous 12 months (see Additional File 2). There was a similar pattern of lower participation and attendance amongst socially disadvantaged groups in the National Taking Part Survey (http://www.culture.gov.uk/what_we_do/research_and_statistics/4828.aspx): levels of both creative activity participation and cultural event attendance were higher amongst employed individuals [creative activities: employed 77.5%, 95% CI 76.5–78.6; unemployed 69.1%, 95% CI 64.0–74.3; cultural events: employed 84.7%, 95% CI 83.8–85.6; unemployed 69.9%, 95% CI 64.6–75.1]. Individuals living in social housing were less likely to participate in creative activities (social housing: 63.4%, 95% CI 61.2–65.6; other accommodation 78.2%, 95% CI 77.3–79.0) or attend cultural events (social housing 59.6%, 95% CI 57.4–61.8; other accommodation 79.6%, 95% CI 78.8–80.4).

### Relationship between creative activity participation/cultural event attendance and health

Creative activity participation and cultural event attendance were both strongly associated with healthy eating and physical activity in the Well London survey population (Table 2 and Table 3). Those who had attended at least one arts event or participated in at least one creative activity in the previous 12 months had approximately twice the odds of meeting the recommended healthy eating (five-a-day) and physical activity (five-a-week) targets. There was also strong evidence that the odds of meeting both these health behaviour recommendations increased with the number of arts events attended and the number of creative activities in which individuals participated.

Creative activity participation and cultural event attendance were associated with positive mental well-being and reduced odds of reporting anxiety or depression in the previous 12 months (Table 3). The associations with both health behaviours and the mental well-being outcomes persisted after adjustment for sociodemographic and economic characteristics, except the association between participation and attendance and self-report anxiety and depression (Table 3).

Adjusting for positive mental well-being or social capital indicators only reduced the odds ratios slightly for the association of participation and attendance with healthy eating and physical activity, and there was still strong statistical support for this association (Table 4). There was evidence of age-specific differences in the relationship between participation in creative activities and healthy eating, physical activity and mental health: the effect of creative activities on meeting the five-a-day and five-a-week targets and on promoting better mental well-being increased with age, except for those aged ≥65 years (Table 5). There was no evidence of statistical interactions for gender, ethnicity or attendance at cultural events. The univariate odds ratios for the sociodemographic characteristics and social capital indicators are shown in Supplementary File 3.

## Discussion

Levels of arts and cultural engagement in low-income groups in London are high, with three-quarters of individuals participating in a creative activity or attending a cultural event at least once a year. However, these levels are still well below the national average. This study found different sociodemographic patterns of arts participation in the Well London population compared with the national Taking Part survey; however, in both surveys, individuals who were more socially disadvantaged (unemployed, living in rented social housing, low educational attainment, low disposable income) were much less likely to have participated. Both participation in creative activities and attendance at cultural events were strongly associated with healthy eating (meeting the five-a-day target), physical activity (meeting the five-a-week target) and positive mental well-being, with no evidence that socio-economic or sociodemographic factors were confounding this relationship. There was no indication that either positive mental well-being or social capital mediate the relationship between arts participation and health behaviours.

This is one of few studies to ask detailed questions specifically about arts and creative activities, rather than general social and cultural participation^20^; by utilizing validated and varied health behaviour and outcome measures.^16–19^ Few studies also distinguish between receptive and active creative or cultural activities,^16,17^ which may plausibly have different mechanisms of effect.^20^ The present authors used the list of arts activities and cultural events, developed by the Department for Culture, Media and Sport and Arts Council for England for the Taking Part survey, as a basis for the arts participation questions in the Well London survey. Whilst some of the activities in Table 1 may not be universally accepted definitions of arts and culture, they were selected for the Taking Part survey using iterative qualitative work with members of the public, policy makers and practitioners, and as such, should be reasonably inclusive and comprehensive.

As with other observational studies, it is possible that reverse causality may operate in the strong association found between arts participation and mental well-being, with individuals who are in better mental health being more disposed to participate in a range of social activities.^39^ However, it seems less plausible that physical activity and healthy eating could be sufficiently important causes of arts participation to explain the strong associations identified in the present study. Whilst adjustment was made for a number of sociodemographic and socio-economic characteristics, it is possible that residual confounding remains. Equally, there is unlikely to be a direct causal link between arts participation and improved health behaviours, although some community-based arts activities may have health themes. It is possible that other mental and cognitive-behavioural factors may confound the relationship, and cause both increased arts participation and better health behaviours. Adjustment for positive mental well-being, using the validated Hope Scale,^25^ did not indicate that this particular facet of mental health was a mediator of the relationship between arts participation and health behaviours, but many other aspects of mental health and cognitive processes, not captured by this scale, could be important. Other authors have suggested that participation in arts activities may reduce mortality because it displaces health-damaging behaviours and activities.^16^

The use of questions that provide more detail about the intensity of arts participation and engagement could have enhanced this study; whilst the authors were able to examine the relationship between the number of different activities or events attended, the frequency of each individual activity was not captured. Therefore, individuals who regularly participate in a single activity would appear to have lower levels of engagement than individuals who participated in three different activities, each on a single occasion, during the 12 month recall period. Therefore, the positive association between the number of arts activities or events and health behaviours should be interpreted with caution.

Whilst this analysis is exploratory and cross-sectional, it supports the calls of other authors for further research in the field of arts and health. Future work could focus on the use of in-depth qualitative methods to understand the processes by which arts participation may impact on mental well-being, health and other behaviours, and on prospective intervention studies to robustly evaluate the health benefits of arts activities in the general population. Finally, if arts are to be commissioned or recommended for health improvement, it will be essential to address the social inequalities in access to arts and cultural activities in order to prevent these from further reinforcing, rather than reducing, health inequalities in the UK.

## Figures and Tables

**Table 1:** Arts and creative events and activities included in the taking part survey question on participation used in the Well London baseline survey.

Creative activities	Cultural events
Dance (any type, not for fitness). Sang to an audience or rehearsed for a performance (not karaoke) or played a musical instrument and/or wrote music. Rehearsed or performed a play (drama, opera/operetta, etc.). Painting, drawing, printmaking or sculpture. Photography or made films or videos as an artistic activity (not family or holiday 'snaps'). Used a computer to create original artwork or animation. Crafts (textile crafts such as embroidery, crocheting or knitting; wood crafts such as wood turning, carving or furniture making; or any other crafts such as calligraphy, pottery or jewellery making). Bought any original/handmade works of art, crafts such as pottery or jewellery for yourself. Read for pleasure (not newspapers, magazines or comics), bought a novel or book of stories, poetry or plays for yourself. Written any stories, plays or poetry. Gone to a nightclub.	Film at a cinema or other venue. Exhibition or collection of art, photography, sculpture or craft exhibition (not craft market). Event that included video or electronic art. Event connected with books or writing. Street arts (art in everyday surroundings such as parks, streets or shopping centres) or circus (not animals). Culturally specific festival (e.g. Carnival, Mela, Baisakhi, Navratri). Any theatre performance (e.g. plays/drama, musical, pantomime). Any music performance such opera/operetta, classical music performance, jazz performance or other live music event. Live dance (such ballet, contemporary dance, ethnic dance performance).

**Table 2:** Prevalence and summary of health behaviours and mental well-being in adults in the Well London baseline survey (based on multiple imputation dataset).

	Participation in creative activities		Attendance at cultural events		Total (95% CI) (*n* = 4107)
No creative activity participation (95% CI) (*n* = 1306)	Creative activity participation (95% CI) (*n* = 2801)	No cultural events attended (95% CI) (*n* = 1621)	Cultural events attended (95% CI) (*n* = 2486)
**Healthy eating**					
Consumption of at least five portions of fruit or vegetables per day (%)	30.4 (26.1–34.7)	40.7 (37.6–43.7)	31.2 (27.7–34.7)	41.5 (38.4–44.6)	37.4 (34.7–40.1)

**Physical activity**					
Five sessions of at least 30 min of moderate-intensity physical activity per week (%)	53.6 (47.3–60.0)	70.2 (66.5–73.9)	53.1 (47.2–59.0)	72.6 (69.0–76.2)	64.9 (60.8–69.0)
MET min per week (mean)	1697 (1292–2102)	2424 (2126–2723)	1619 (1271–1967)	2567 (2261–2874)	2193 (1884–2503)

**Mental health**					
Hope Scale score^a^ (mean)	4.4 (4.3–4.5)	4.7 (4.6–4.7)	4.4 (4.3–4.5)	4.7 (4.6–4.7)	4.6 (4.5–4.6)
Self-report feeling anxious or depressed (EuroQol 5D) (%)	22.7 (18.7–26.6)	16.2 (13.0–19.4)	22.6 (19.0–26.3)	15.4 (12.1–18.7)	18.3 (15.1–21.4)
Consult general practitioner for anxiety/depression/emotional problems	18.3 (16.7–23.9)	15.7 (12.1–19.2)	18.8 (13.8–23.9)	15.0 (11.3–18.6)	16.5 (12.6–20.4)

CI, confidence interval; MET, metabolic equivalent of task.

a Higher score indicates greater hopefulness; maximum score 48 (collected using six-point Likert scale responses).

**Table 3:** Univariate and adjusted odds ratios and regression coefficients for arts participation and health behaviours and mental well-being in adults from the Well London survey (adjustment for sociodemographic factors; based on multiple imputation analysis; *n* = 4107).

	Healthy eating		Physical activity		Mental health			
Consumption of at least five portions of fruit or vegetables per day (%)		Five sessions of at least 30 min of moderate-intensity physical activity per week (%)		Hope scale score^a^		Self-report feeling anxious or depressed (EuroQol 5D)	
Or (95% CI)	Wald test *P*-value	Or (95% CI)	Wald test *P*-value	Coeff (95% CI)	Wald test *P*-value	Or (95% CI)	Wald test *P*-value
Univariate effect estimates								
Creative activity participation	1.7 (1.4–1.9)	<0.001	2.1 (1.8–2.5)	<0.001	0.29 (0.24–0.34)	<0.001	0.6 (0.5–0.7)	<0.001
Number of creative activities	1.1 (1.1–1.2)	<0.001	1.3 (1.2–1.3)	<0.001	0.07 (0.06–0.08)	<0.001	0.9 (0.9–1.0)	0.002
Cultural event attendance	1.7 (1.4–1.9)	<0.001	2.6 (2.3–3.0)	<0.001	0.27 (0.22–0.31)	<0.001	0.6 (0.5–0.7)	<0.001
Number of cultural events	1.2 (1.1–1.2)	0.01	1.4 (1.3–1.4)	<0.001	0.08 (0.06–0.09)	<0.001	0.9 (0.9–0.9)	<0.001
Adjusted effect estimates – Sociodemographics^b^								
Creative activity participation	1.8 (1.6–2.2)	<0.001	1.7 (1.4–2.0)	<0.001	0.2 (0.2–0.3)	<0.001	0.9 (0.8–1.2)	0.56
Number of creative activities	1.1 (1.1–1.2)	<0.001	1.2 (1.2–1.3)	<0.001	0.05 (0.04–0.06)	<0.001	1.1 (1.0–1.1)	0.02
Cultural event attendance	1.8 (1.6–2.2)	<0.001	2.1 (1.8–2.4)	<0.001	0.2 (0.1–0.2)	<0.001	0.9 (0.8–1.1)	0.52
Number of cultural events	1.2 (1.2–1.3)	<0.001	1.3 (1.2–1.3)	<0.001	0.05 (0.04–0.07)	<0.001	1.0 (1.0–1.1)	0.12

OR, odds ratio; Coef, linear regression coefficient; CI, confidence interval.

a Higher score indicates greater hopefulness; maximum score 48 (collected using six-point Likert scale responses).

b Effect estimates adjusted for age, gender, ethnicity, employment status, housing tenure, ease of managing on household income, and educational attainment.

**Table 4:** Adjusted odds ratios and regression coefficients for arts participations and health behaviours in adults from the Well London survey (adjustment for mental well-being and social capital; based on multiple imputation analysis; *n* = 4107).

	Healthy eating		Physical activity	
Consumption of at least five portions of fruit or vegetables per day (%)		Five sessions of at least 30 min of moderate-intensity physical activity per week (%)	
Or (95% CI)	Wald test *P*-value	Or (95% CI)	Wald test *P*-value
Adjusted effect estimates – Sociodemographics^b^ hope scale^a^				
Creative activity participation	1.7 (1.5–2.0)	<0.001	1.6 (1.4–1.9)	<0.001
Number of creative activities	1.1 (1.1–1.2)	<0.001	1.2 (1.1–1.3)	<0.001
Cultural event attendance	1.8 (1.5–2.1)	<0.001	2.0 (1.7–2.3)	<0.001
Number of cultural events	1.2 (1.2–1.3)	<0.001	1.2 (1.2–1.3)	<0.001
Adjusted effect estimates – Sociodemographics^b^ social capital				
Creative activity participation	1.9 (1.6–2.2)	0.02	1.6 (1.4–1.9)	<0.001
Number of creative activities	1.1 (1.1–1.2)	<0.001	1.2 (1.1–1.3)	<0.001
Cultural event attendance	1.9 (1.6–2.2)	<0.001	2.0 (1.7–2.3)	<0.001
Number of cultural events	1.2 (1.2–1.3)	<0.001	1.3 (1.2–1.3)	<0.001

OR, odds ratio; Coef, linear regression coefficient; CI, confidence interval.

a Higher score indicates greater hopefulness; maximum score 48 (collected using six-point Likert scale responses).

b Effect estimates adjusted for age, gender, ethnicity, employment status, housing tenure, ease of managing on household income, and educational attainment.

**Table 5:** Age-specific odds ratios for the effect of participation in creative activities on healthy eating, physical activity and mental health and well-being in adults from the Well London survey (based on multiple imputation analysis; *n* = 4107).

	Healthy eating		Physical activity		Mental health			
Consumption of at least five portions of fruit or vegetables per day (%)		Five sessions of at least 30 min of moderate-intensity physical activity per week (%)		Hope scale score^a^		Self-report feeling anxious or depressed (EuroQol 5D)	
Or (95% CI)	Likelihood ratio test *P*-value	Or (95% CI)	Likelihood ratio test *P*-value	Coeff (95% CI)	Likelihood ratio test *P*-value	Or (95% CI)	Likelihood ratio test *P*-value
Creative activity participation								
16–24 years	1.3 (0.9–2.1)	0.2	1.3 (0.8–2.1)	0.02	0.16 (0.02–0.30)	0.007	1.4 (0.7–2.8)	0.03
25–34 years	1.6 (1.2–2.1)		1.7 (1.3–2.3)		0.19 (0.09–0.29)		0.8 (0.6–1.2)	
35–44 years	1.5 (1.1–2.1)		1.8 (1.3–2.5)		0.28 (0.18–0.39)		0.8 (0.6–1.2)	
45–54 years	2.3 (1.1–3.5)		2.9 (1.9–4.5)		0.45 (0.31–0.60)		0.6 (0.4–0.9)	
55–64 years	2.4 (1.4–4.0)		3.2 (1.9–5.3)		0.35 (0.17–0.52)		0.3 (0.2–0.6)	
≥65 years	2.2 (1.4–3.5)		1.4 (0.9–2.1)		0.44 (0.28–0.59)		0.9 (0.6–1.5)	

OR, odds ratio; Coef, linear regression coefficient; CI, confidence interval.

a Higher score indicates greater hopefulness; maximum score 48 (collected using six-point Likert scale responses).
