# Supplementary material for: Think of your art-eries: Arts participation, behavioural cardiovascular risk factors and mental well-being in deprived communities in London
Source: Public Health. 2012 Sep 1;126(5):S57–64. doi: 10.1016/j.puhe.2012.05.025 (PMC3449238; doi:10.1016/j.puhe.2012.05.025)
Supplement: Supplementary file 1 [file mmc1.docx]

Table : Missing data in regression variables from the *Well London* adult survey

| Variable | Percent responses missing |
| --- | --- |
| Age | 9.2 |
| Gender | 1.8 |
| Ethnicity | 2.5 |
| Employment status | 6.7 |
| Educational achievement | 11.1 |
| Housing tenure | 6.1 |
| Ease of managing on household income | 6.8 |
| Social capital |  |
| Meet with friends at least once a week | 1.6 |
| Speak to friends on the telephone at least once a week | 1.9 |
| Write to friends (letters, texting, emails, internet) at least once a week | 3.0 |
| Speak to neighbours at least once a week | 2.7 |
| Number of people who would provide help with groceries if unwell | 3.9 |
| Number of people who would lend money for a few days | 5.6 |
| Number of people who would give advice and support in a crisis | 4.7 |
| Healthy eating (portions of fruit and vegetables per day | 11.9 |
| Physical activity (MET minutes per week) | 14.1 |
| Mental health |  |
| Hope scale | 11.0 |
| Self-report anxious or depressed (EQ5D) | 2.7 |
| Consult GP for anxiety/ depression/ emotional or nervous problem | 3.9 |
