# Supplementary material for: Think of your art-eries: Arts participation, behavioural cardiovascular risk factors and mental well-being in deprived communities in London
Source: Public Health. 2012 Sep 1;126(5):S57–64. doi: 10.1016/j.puhe.2012.05.025 (PMC3449238; doi:10.1016/j.puhe.2012.05.025)
Supplement: Supplementary file 2 [file mmc2.docx]

Table : Sociodemographic characteristics of adults from the *Well London* survey (based on multiply imputed dataset)

|  | No creative activity participation  (95% CI)  (n=1306) | Creative activity participation  (95% CI)  (n=2801) | No cultural events attended  (95% CI)  (n=1621) | Cultural events attended  (95% CI)  (n=2486) | Total  (95% CI)  (n=4107) |
| --- | --- | --- | --- | --- | --- |
| Age in years % |  |  |  |  |  |
| 16 – 24 | 10.3 (8.0, 12.7) | 26.3 (23.8, 28.8) | 9.4 (7.4, 11.5) | 28.9 (26.5, 31.4) | 21.2 (19.4, 23.0) |
| 25 – 34 | 24.9 (21.4, 28.5) | 27.5 (24.8, 30.2) | 22.3 (19.3, 25.3) | 29.6 (26.4, 32.7) | 26.7 (24.1, 29.3) |
| 35 – 44 | 24.7 (21.4, 27.9) | 22.9 (20.5, 25.4) | 23.0 (19.9, 26.1) | 23.8 (21.4, 26.3) | 23.5 (21.4, 25.6) |
| 45 – 54 | 13.5 (11.6, 15.4) | 11.3 (9.8, 12.9) | 15.9 (13.8, 18.0) | 9.5 (8.0, 11.0) | 12.0 (10.7, 13.4) |
| 55 – 64 | 11.1 (9.3, 13.0) | 5.8 (4.5, 7.1) | 12.1 (10.3, 14.0) | 4.5 (3.4, 5.6) | 7.5 (6.3, 8.7) |
| 65+ | 15.4 (11.9, 18.9) | 6.1 (4.7, 7.4) | 17.2 (13.9, 20.5) | 3.7 (2.7, 4.8) | 9.0 (7.3, 10.8) |
| Gender % |  |  |  |  |  |
| Females | 54.4 (51.0, 57.8) | 55.5 (53.0, 58.0) | 56.8 (53.9, 59.7) | 54.1 (51.2, 57.0) | 55.2 (52.9, 57.5) |
| Ethnicity % |  |  |  |  |  |
| White British | 28.4 (23.1, 33.7) | 32.1 (26.9, 37.3) | 31.4 (25.5, 37.3) | 30.6 (25.5, 35.7) | 30.9 (26.0, 35.9) |
| White Other | 11.3 (8.4, 14.3) | 14.2 (11.2, 17.2) | 11.3 (8.5, 14.2) | 14.5 (11.4, 17.6) | 13.3 (10.6, 15.9) |
| Black Caribbean | 9.7 (7.1, 12.3) | 12.8 (10.3, 15.2) | 10.6 (7.9, 13.4) | 12.5 (10.0, 45.0) | 11.8 (9.5, 14.0) |
| Black African | 15.5 (11.6, 19.6) | 17.3 (13.6, 20.9) | 17.8 (13.7, 21.8) | 16.0 (12.2, 19.9) | 16.7 (13.3, 20.2) |
| Indian/ Pakistani/ Bangladeshi | 15.1 (7.4, 22.8) | 8.4 (4.3, 12.4) | 12.7 (6.0, 19.4) | 9.1 (5.0, 13.1) | 10.5 (5.7, 15.3) |
| Other Asian | 5.4 (3.6, 7.2) | 4.0 (2.6, 5.4) | 4.1 (2.8, 5.3) | 4.7 (2.9, 6.5) | 4.5 (3.1, 5.9) |
| Mixed ethnicity | 3.3 (1.9, 4.7) | 5.4 (4.3, 6.5) | 2.8 (1.7, 4.0) | 6.0 (4.7, 7.3) | 4.8 (3.8, 5.8) |
| Other | 11.2 (6.7, 15.8) | 5.9 (3.9, 7.9) | 9.2 (5.5, 12.8) | 6.5 (4.6, 8.4) | 7.6 (5.2, 10.0) |
| Employed full or part time % | 28.0 (24.0, 32.1) | 49.3 (46.0, 52.5) | 29.9 (25.7, 34.1) | 50.7 (47.2, 54.3) | 42.5 (39.2, 45.8) |
| Educational level achieved % |  |  |  |  |  |
| No qualifications | 21.2 (15.4, 27.0) | 5.4 (3.8, 6.9) | 19.6 (14.1, 25.0) | 4.5 (3.0, 6.1) | 10.4 (7.4, 13.4) |
| GCSE or equivalent | 36.9 (32.4, 41.5) | 30.9 (27.2, 34.6) | 37.8 (33.0, 42.7) | 29.6 (26.1, 33.2) | 32.8 (29.4, 36.3) |
| A-level or equivalent | 24.2 (20.2, 28.2) | 30.4 (28.1, 32.7) | 24.4 (20.7, 28.1) | 30.9 (28.5, 33.4) | 28.4 (26.0, 30.8) |
| University degree | 16.8 (13.5, 20.2) | 32.3 (28.4, 36.2) | 17.4 (14.0, 20.7) | 33.9 (29.6, 38.1) | 27.4 (23.9, 30.8) |
| Other | 0.8 (0.2, 1.5) | 1.0 (0.3, 1.8) | 0.8 (0.2, 1.5) | 1.0 (0.4, 1.7) | 1.0 (0.4, 1.6) |
| Housing tenure |  |  |  |  |  |
| Rent – social housing | 61.7 (52.9, 70.5) | 49.8 (43.3, 56.4) | 64.6 (56.9, 72.4) | 46.4 (39.5, 53.3) | 53.6 (46.8, 60.4) |
| Rent/ mortgage | 0.9 (0.3, 1.5) | 1.7 (0.8, 2.7) | 0.9 (0.3, 1.5) | 1.8 (0.8, 2.9) | 1.5 (0.8, 2.2) |
| Owner occupier | 10.2 (7.1, 13.2) | 15.4 (12.7, 18.0) | 11.4 (8.2, 14.5) | 15.2 (12.6, 17.9) | 13.7 (11.3, 16.1) |
| Rent – private landlord | 9.6 (6.1, 13.1) | 14.5 (10.9, 18.2) | 8.6 (5.6, 11.6) | 15.8 (11.8, 19.8) | 13.0 (9.8, 16.2) |
| Other | 17.8 (10.2, 25.3) | 18.5 (13.9, 23.1) | 14.5 (8.2, 20.8) | 20.7 (15.7, 25.7) | 18.3 (13.2, 23.4) |
| Ease of managing on household income % |  |  |  |  |  |
| Very difficult | 32.5 (25.3, 39.8) | 20.4 (16.9, 23.9) | 30.1 (23.7, 36.5) | 20.4 (16.7, 24.1) | 24.3 (19.7, 28.8) |
| Fairly difficult | 24.3 (19.3, 29.3) | 26.8 (22.0, 31.6) | 25.5 (20.6, 30.4) | 26.3 (21.6, 31.2) | 26.0 (21.6, 30.5) |
| Neither easy nor difficult | 26.6 (21.0, 32.2) | 30.2 (25.0, 35.4) | 27.3 (21.8, 32.8) | 30.2 (24.9, 35.4) | 29.0 (23.9, 34.1) |
| Fairly easy | 14.7 (11.3, 18.1) | 18.7 (15.2, 22.1) | 15.4 (12.1, 18.7) | 18.8 (15.2, 22.3) | 17.4 (14.3, 20.6) |
| Very easy | 1.8 ( 0.9, 2.7) | 3.9 (2.8, 5.1) | 1.7 (1.0, 2.4) | 4.3 (3.0, 5.6) | 3.3 (2.4, 4.1) |
| Social capital |  |  |  |  |  |
| Meet with friends at least once a week | 71.7 (67.3, 76.2) | 79.7 (76.5, 82.9) | 70.8 (67.2, 74.5) | 81.3 (77.8, 84.8) | 77.2 (74.1, 80.3) |
| Speak to friends on the telephone at least once a week | 81.3 (77.3, 85.3) | 88.6 (86.4, 90.9) | 80.3 (76.5, 84.0) | 90.2 (88.1, 92.4) | 86.3 (84.0, 88.6) |
| Write to friends (letters, texting, emails, internet) at least once a week | 48.4 (39.9, 56.9) | 65.6 (60.4, 70.9) | 47.1 (39.2, 54.9) | 68.7 (63.2, 74.1) | 60.1 (55.0, 65.3) |
| Speak to neighbours at least once a week | 68.7 (62.5, 69.4) | 63.9 (58.4, 69.4) | 67.9 (62.3, 73.5) | 63.8 (57.6, 70.0) | 65.4 (60.1, 70.7) |
| Number of people who would provide help with groceries if unwell |  |  |  |  |  |
| None | 22.0 (17.9, 26.0) | 15.6 (12.3, 18.9) | 21.1 (17.4, 24.9) | 15.3 (12.0, 18.6) | 17.6 (14.5, 20.7) |
| One or two | 51.8 (47.3, 56.3) | 46.6 (43.2, 50.1) | 51.6 (46.8, 56.3) | 46.1 (43.0, 49.3) | 48.3 (45.0, 51.6) |
| More than two | 21.5 (17.2, 25.8) | 32.4 (28.3, 36.4) | 22.8 (18.6, 27.0) | 32.9 (28.9, 36.9) | 28.9 (25.2, 32.7) |
| Would not ask | 4.7 (2.0, 7.4) | 5.4 (3.6, 7.2) | 4.5 (2.0, 7.0) | 5.6 (3.7, 7.5) | 5.2 (3.3, 7.1) |
| Number of people who would lend money for a few days |  |  |  |  |  |
| None | 24.7 (19.7, 29.7) | 14.6 (11.7, 17.5) | 24.6 (20.1, 29.1) | 13.4 (10.5, 16.2) | 17.8 (14.5, 21.1) |
| One or two | 42.5 (38.1, 47.0) | 39.5 (36.3, 42.7) | 40.4 (36.2, 44.7) | 40.5 (37.1, 43.9) | 40.5 (37.3, 43.6) |
| More than two | 17.2 (12.9, 21.6) | 28.5 (24.8, 32.2) | 17.6 (13.6, 21.5) | 29.7 (25.9, 33.5) | 24.9 (21.4, 28.5) |
| Would not ask | 51.6 (11.1, 19.9) | 17.4 (14.2, 20.7) | 17.5 (13.2, 21.7) | 16.4 (13.2, 19.6) | 16.8 (13.5, 20.1) |
| Number of people who would give advice and support in a crisis |  |  |  |  |  |
| None | 21.8 (16.9, 26.6) | 10.8 (8.2, 13.5) | 21.3 (17.0, 25.5) | 9.8 (7.2, 12.3) | 14.3 (11.2, 17.4) |
| One or two | 50.8 (46.2, 55.5) | 44.0 (40.5, 47.4) | 49.5 (45.3, 53.7) | 43.9 (40.2, 47.7) | 46.2 (42.8, 49.5) |
| More than two | 21.8 (17.0, 26.5) | 39.8 (35.6, 43.9) | 23.6 (19.1, 28.1) | 40.8 (36.4, 45.2) | 34.0 (30.0, 3.1) |
| Would not ask | 5.7 (3.3, 8.0) | 5.4 (3.7, 7.1) | 5.6 (3.3, 7.9) | 5.4 (3.7, 7.1) | .5 (3.7, 7.3) |

^a^ Higher score indicates greater hopefulness; maximum score 48 (collected using 6-point likert scale responses).

Abbreviations: OR, odds ratio; Coef, linear regression coefficient; CI, confidence interval.
