# Supplementary material for: Think of your art-eries: Arts participation, behavioural cardiovascular risk factors and mental well-being in deprived communities in London
Source: Public Health. 2012 Sep 1;126(5):S57–64. doi: 10.1016/j.puhe.2012.05.025 (PMC3449238; doi:10.1016/j.puhe.2012.05.025)
Supplement: Supplementary file 3 [file mmc3.docx]

Table : Univariate odds ratios and regression coefficients for sociodemographic characteristics and arts participations in adults (using multiply imputed datasets, n=4107)

|  | Healthy eating | | Physical activity | | Mental Health | | | | | |
| --- | --- | --- | --- | --- | --- | --- | --- | --- | --- | --- |
|  | Meeting five-a-day | | meeting 5x30mins per week | | Hope Scale score ^a^ | | Self-report feeling anxious or depressed (EQ5D) | | Consult GP for anxiety/ depression/ emotional or nervous problem | |
|  | OR  (95% CI) | Overall Wald test *P* value | OR  (95% CI) | Overall Wald test *P* value | Coeff (95% CI) | Overall Wald test *P* value | OR  (95% CI) | Overall Wald test *P* value | OR  (95% CI) | Overall Wald test *P* value |
| Age in years |  | 0.04 |  | <0.001 |  | <0.001 |  | <0.001 |  | <0.001 |
| 16 – 24 | 1.0 |  | 1.0 |  | - |  | 1.0 |  | 1.0 |  |
| 25 – 34 | 1.3 (1.1, 1.6) |  | 0.8 (0.6, 0.9) |  | -0.01  (-0.08, 0.06) |  | 1.5 (1.1, 2.0) |  | 1.3 (1.0, 1.8) |  |
| 35 – 44 | 1.1 (0.9, 1.4) |  | 0.6 (0.5, 0.7) |  | 0.02  (-0.05, 0.09) |  | 2.4 (1.8, 3.2) |  | 1.7 (1.3, 2.4) |  |
| 45 – 54 | 1.4 (1.1, 1.8) |  | 0.6 (0.5, 0.8) |  | -0.13  (-0.21, -0.04) |  | 3.9 (2.9, 5.4) |  | 3.0 (2.2, 4.1) |  |
| 55 – 64 | 1.2 (0.9, 1.6) |  | 0.4 (0.3, 0.6) |  | -0.18  (-0.28, -0.08) |  | 4.3 (3.0, 6.1) |  | 3.2 (2.2, 4.5) |  |
| 65+ | 1.0 (0.8, 1.3) |  | 0.3 (0.2, 0.3) |  | -0.10  (-0.20, -0.01) |  | 3.6 (2.6, 5.0) |  | 2.3 (1.6, 3.3) |  |
| Gender |  | 0.58 |  | <0.001 |  | 0.28 |  | 0.45 |  | 0.005 |
| Males | 1.0 |  | 1.0 |  | - |  | 1.0 |  | 1.0 |  |
| Females | 1.0 (0.9, 1.2) |  | 0.6 (0.6, 0.7) |  | -0.03  (-0.07, 0.02) |  | 1.1 (0.9, 1.3) |  | 1.3 (1.1, 1.6) |  |
| Ethnicity |  | <0.001 |  | 0.002 |  |  |  | <0.001 |  | 0.01 |
| White British | 1.0 |  | 1.0 |  | - | <0.001 | 1.0 |  | 1.0 |  |
| White Other | 1.9 (1.5, 2.3) |  | 1.2 (0.9, 1.5) |  | 0.08  (0.00, 0.16) |  | 0.7 (0.5, 0.9) |  | 0.7 (0.5, 0.9) |  |
| Black Caribbean | 0.9 (0.7, 1.2) |  | 0.8 (0.7, 1.0) |  | 0.07  (-0.01, 0.15) |  | 0.6 (0.4, 0.8) |  | 0.7 (0.5, 1.0) |  |
| Black African | 1.2 (1.0, 1.4) |  | 1.0 (0.8, 1.2) |  | 0.19  (0.11, 0.26) |  | 0.4 (0.3, 0.6) |  | 0.6 (0.5, 0.9) |  |
| Indian/ Pakistani/ Bangladeshi | 1.6 (1.2, 2.1) |  | 1.0 (0.7, 1.3) |  | 0.05  (-0.04, 0.15) |  | 0.5 (0.3, 0.7) |  | 0.6 (0.4, 0.9) |  |
| Other Asian | 1.5 (1.1, 2.1) |  | 0.6 (0.4, 0.8) |  | 0.16  (0.03, 0.28) |  | 0.7 (0.5, 1.1) |  | 0.8 (0.5, 1.3) |  |
| Mixed ethnicity | 1.1 (0.8, 1.5) |  | 1.4 (1.0, 1.9) |  | 0.13  (0.02, 0.25) |  | 0.5 (0.3, 0.8) |  | 0.7 (0.4, 1.0) |  |
| Other | 1.7 (1.3, 2.2) |  | 0.9 (0.7, 1.2) |  | 0.10  (0.00, 0.20) |  | 0.6 (0.4, 0.8) |  | 0.6 (0.4, 0.9) |  |
| Employment status |  | <0.001 |  | <0.001 |  | <0.001 |  | <0.001 |  | <0.001 |
| Employed full or part time | 1.0 |  | 1.0 |  | - |  | 1.0 |  | 1.0 |  |
| Unemployed looking for work | 0.7 (0.6, 0.8) |  | 0.6 (0.5, 0.7) |  | -0.45  (-0.50, -0.38) |  | 3.1 (2.4, 3.8) |  | 2.2 (1.7, 2.8) |  |
| Housework/ education/ retired/ ill/ other | 0.8 (0.7, 0.9) |  | 0.5 (0.4, 0.5) |  | -0.24  (-0.29, -0.18) |  | 2.5 (2.0, 3.1) |  | 1.9 (0.6, 2.4) |  |
| Housing tenure |  | <0.001 |  | <0.001 |  | <0.001 |  | <0.001 |  | <0.001 |
| Rent – social housing | 1.0 |  | 1.0 |  | - |  | 1.0 |  | 1.0 |  |
| Rent/ mortgage | 1.1 (0.6, 1.9) |  | 1.6 (0.9, 3.0) |  | 0.15  (-0.06, 0.37) |  | 0.5 (0.2, 1.2) |  | 0.2 (0.07, 0.8) |  |
| Owner occupier | 1.3 (1.1, 1.6) |  | 1.2 (1.0, 1.5) |  | 0.28  (0.20, 0.35) |  | 0.6 (0.5, 0.8) |  | 0.6 (0.5, 0.9) |  |
| Rent – private landlord | 1.5 (1.2, 1.9) |  | 1.9 (1.5, 2.4) |  | 0.18  (0.10, 0.25) |  | 0.6 (0.5, 0.8) |  | 0.4 (0.3, 0.6) |  |
| Other | 1.1 (0.9, 1.3) |  | 1.3 (1.1, 1.6) |  | -0.01  (-0.08, 0.06) |  | 0.6 (0.5, 0.8) |  | 0.5 (0.4, 0.7) |  |
| Ease of managing on household income |  | 0.31 |  | 0.42 |  | <0.001 |  | <0.001 |  | <0.001 |
| Very difficult | 1.0 |  | 1.0 |  | - |  | 1.0 |  | 1.0 |  |
| Fairly difficult | 1.0 (0.8, 1.2) |  | 1.0 (0.8, 1.3) |  | 0.08  (0.02, 0.16) |  | 0.7 (0.5, 0.9) |  | 0.8 (0.6, 1.0) |  |
| Neither easy nor difficult | 1.1 (0.9, 1.4) |  | 1.1 (0.8, 1.3) |  | 0.30  (0.23, 0.37) |  | 0.4 (0.3, 0.5) |  | 0.4 (0.3, 0.6) |  |
| Fairly easy | 1.2 (1.0, 1.5) |  | 1.2 (0.9, 1.5) |  | 0.27  (0.19, 0.34) |  | 0.4 (0.3, 0.5) |  | 0.6 (0.5, 0.9) |  |
| Very easy | 0.9 (0.6, 1.3) |  | 1.6 (1.0, 2.4) |  | 0.48  (0.34, 0.62) |  | 0.4 (0.2, 0.7) |  | 0.7 (0.4, 1.2) |  |
| Social capital |  |  |  |  |  |  |  |  |  |  |
| Meet with friends at least once a week | 1.0 (0.9, 1.2) | 0.96 | 1.2 (1.5, 2.0) | <0.001 | 0.26  (0.20, 0.31) | <0.001 | 0.4 (0.4, 0.5) | <0.001 | 0.5 (0.4, 0.6) | <0.001 |
| Speak to friends on the telephone at least once a week | 1.2 (1.0, 1.4) | 0.09 | 1.8 (1.5, 2.2) | <0.001 | 0.37  (0.31, 0.44) | <0.001 | 0.4 (0.3, 0.5) | <0.001 | 0.5 (0.4, 0.7) | <0.001 |
| Write to friends (letters, texting, emails, internet) at least once a week | 1.1 (0.9, 1.2) | 0.32 | 1.7 (1.5, 2.0) | <0.001 | 0.27  (0.22, 0.32) | <0.001 | 0.4 (0.3, 0.5) | <0.001 | 0.5 (0.4, 0.6) | <0.001 |
| Speak to neighbours at least once a week | 1.3 (1.1, 1.5) | <0.001 | 1.1 (1.0, 1.3) | 0.20 | 0.11  (0.06, 0.16) | <0.001 | 1.0 (0.8, 1.2) | 0.04 | 1.1 (0.9, 1.3) | 0.32 |
| Number of people who would provide help with groceries if unwell |  | 0.02 |  | 0.06 |  | <0.001 |  | <0.001 |  | 0.1 |
| None | 1.0 |  | 1.0 |  | - |  | 1.0 |  | 1.0 |  |
| One or two | 0.8 (0.7, 1.0) |  | 1.0 (0.8, 1.2) |  | 0.16  (0.09, 0.23 |  | 1.0 (0.8, 1.3) |  | 0.9 (0.7, 1.2) |  |
| More than two | 1.1 (0.9, 1.3) |  | 1.3 (1.0, 1.6) |  | 0.31  (0.23, 0.38) |  | 0.6 (0.5, 0.8) |  | 0.7 (0.6, 1.0) |  |
| Would not ask | 1.0 (0.7, 1.4) |  | 1.1 (0.8, 1.6) |  | -0.04  (-0.16, 0.09) |  | 0.9 (0.6, 1.4) |  | 0.8 (0.5, 1.3) |  |
| Number of people who would lend money for a few days |  | 0.009 |  | <0.001 |  | <0.001 |  | <0.001 |  | <0.001 |
| None | 1.0 |  | 1.0 |  | - |  | 1.0 |  | 1.0 |  |
| One or two | 0.8 (0.7, 1.0) |  | 1.1 (0.9, 1.4) |  | 0.09  (0.02, 0.16) |  | 0.8 (0.6, 1.0) |  | 0.9 (0.7, 1.2) |  |
| More than two | 1.1 (0.9, 1.3) |  | 1.6 (1.3, 2.0) |  | 0.31  (0.24, 0.40) |  | 0.5 (0.4, 0.6) |  | 0.5 (0.4, 0.7) |  |
| Would not ask | 0.9 (0.7, 1.2) |  | 1.3 (1.0, 1.7) |  | 0.14  (0.06, 0.23) |  | 0.7 (0.5, 0.9) |  | 0.9 (0.7, 1.2) |  |
| Number of people who would give advice and support in a crisis |  | 0.19 |  | <0.001 |  | <0.001 |  | <0.001 |  | 0.01 |
| None | 1.0 |  | 1.0 |  | - |  | 1.0 |  | 1.0 |  |
| One or two | 0.8 (0.7, 1.1) |  | 1.2 (1.0, 1.4) |  | 0.14  (0.07, 0.21) |  | 0.9 (0.7, 1.2) |  | 1.1 (0.8, 1.4) |  |
| More than two | 1.1 (0.9, 1.3) |  | 1.7 (1.3, 2.1) |  | 0.32  (0.24, 0.40) |  | 0.6 (0.5, 0.8) |  | 0.7 (0.6, 1.0) |  |
| Would not ask | 1.1 (0.7, 1.5) |  | 1.4 (1.0, 2.0) |  | 0.09  (-0.04, 0.21) |  | 0.7 (0.5, 1.1) |  | 0.9 (0.6, 1.5) |  |
| Arts participation | 1.7 (1.4, 1.9) | <0.001 | 2.1 (1.8, 2.5) | <0.001 | 0.29  (0.24, 0.34) | <0.001 | 0.6 (0.5, 0.7) | <0.001 | 0.7 (0.6, 0.9) | 0.002 |
| Number of arts activities participated | 1.1 (1.1, 1.2) | <0.001 | 1.3 (1.2, 1.3) | <0.001 | 0.07  (0.06, 0.08) | <0.001 | 0.9 (0.9, 1.0) | 0.002 | 1.0 (0.9, 1.0) | 0.1 |
| Arts spectator | 1.7 (1.4, 1.9) | <0.001 | 2.6 (2.3, 3.0) | <0.001 | 0.27  (0.22, 0.31) | <0.001 | 0.6 (0.5, 0.7) | <0.001 | 0.7 (0.6, 0.8) | <0.001 |
| Number of arts events attended | 1.2 (1.1, 1.2) | 0.01 | 1.4 (1.3, 1.4) | <0.001 | 0.08  (0.06, 0.09) | <0.001 | 0.9 (0.9, 0.9) | <0.001 | 1.0 (0.9, 1.0) | 0.1 |

^a^ Higher score indicates greater hopefulness; maximum score 48 (delivered using 6-point likert scale responses).

Abbreviations: OR, odds ration; Coef, linear regression coefficient; CI, confidence interval.
